# Polymorphisms of stress pathway genes and emergence of suicidal ideation at antidepressant treatment onset

**DOI:** 10.1038/s41398-020-01003-0

**Published:** 2020-09-20

**Authors:** B. Nobile, N. Ramoz, I. Jaussent, J. Dubois, S. Guillaume, Ph Gorwood, Ph Courtet

**Affiliations:** 1 Department of Emergency Psychiatry and Acute Care, Lapeyronie Hospital, CHU Montpellier, Montpellier, France; 2 PSNREC, Univ Montpellier, INSERM, CHU de Montpellier, Montpellier, France; 3 Inserm UMRS1266, Institute of Psychiatry and Neuroscience of Paris, Paris, France; 4 FondaMental Foundation, Créteil, France

**Keywords:** Clinical genetics, Neuroscience, Psychiatric disorders

## Abstract

The prescription of antidepressant drugs is one of the most frequently used strategies to prevent suicide and suicidal behavior. However, some patients develop suicidal ideation at antidepressant treatment onset, a phenomenon known as treatment-emergent suicidal ideation (TESI). Few studies have explored TESI pharmacogenomics. As the Hypothalamic-Pituitary-Adrenal (HPA) axis might be implicated in suicidal behavior, we assessed the relationship between TESI and single nucleotide polymorphisms (SNPs) in the HPA axis-implicated *NR3C1* (*n* = 7 SNPs), *FKBP5* (*n* = 5 SNPs), *AVPR1B* (*n* = 1 SNPs), *CRHR1* (*n* = 1 SNPs), and *SKA2* (*n* = 1 SNPs) genes, in a sample of 3566 adult outpatients with depression for whom an antidepressant treatment was introduced. General practitioners and psychiatrists throughout France followed participants for 6 weeks after the initial prescription of tianeptine, an antidepressant molecule showing mu agonism. Suicidal ideation was assessed with item 10 of the Montgomery-Åsberg Depression Rating Scale (item dedicated to suicidal ideation) at baseline, and at week 2, 4, and 6 of treatment. Within the informative sample, 112 patients reported TESI and 384 did not. TESI was significantly associated with the TT genotype of the SNP rs6902321 in *FKBP5* (OR = 1.76, 95% CI = [1.07; 2.90]; *p*-value = 0.03) and the GG/AG genotype of the SNP rs7208505 in *SKA2* (OR = 1.85, 95% CI = [1.03;3.33]; *p*-value = 0.04). These associations were not significant after multiple test correction. Nevertheless, our results suggest a possible involvement of HPA axis elements in treatment-emergent suicidal ideation (TESI).

## Introduction

Every year, more than 800,000 persons die by suicide in the world and the number of suicide attempts (SA) is even higher, according to the World Health Organization. About 90% of suicide completers have a psychiatric problems, mostly major depressive disorder (MDD)^1^. This suggests that suicidal behavior (SB) is linked to mental disorders. Nevertheless, SB has been entered in the DSM-5, independently of other pathologies, implying its own physiopathology. Although not part of the SBs included in the DSM-5, suicidal ideation (SI) is the third most important risk factor of death by suicide^2^, and targeting SI to reduce SB seems a good strategy. Growing evidences suggest that SI course is related to, but separated from changes in depressive symptomatology, implying that different mechanisms underlie these changes^3^. Indeed, there is no evidence that SB will disappear once the psychiatric disorder is well managed. This suggests that patients with SB might need specific treatments^4^. However, SB physiopathology remains unclear, and no specific SB biomarker is available, making it difficult to detect persons at risk and to prevent SA or completed suicide. In the last decade, much effort has been directed to prevent suicide and SA, for instance by reducing access to lethal means (i.e. control of analgesics use), by prevention programs in school, and by using clozapine and lithium and psychological therapies^5^. Actually, one of the most frequent strategies to prevent suicide and SB is antidepressant prescription^6^. Yet, an international controversy began in the 1990s concerning antidepressant use and treatment emergence or worsening of suicidal ideation (TESI/TWOSI). This led to the application of a black box warning on antidepressant use by the US Food and Drug Administration (FDA)^7^. However, it has been estimated that if all patients with depression received antidepressants, more than one in three suicide deaths would be prevented compared with no antidepressant prescription^8^. Consequently, it would be useful to find TESI/TWOSI biomarkers to identify patients at risk of TESI/TWOSI rather than not prescribing antidepressants. Some clinical biomarkers have been identified in clinical studies on TESI and TWOSI (i.e., pre-adult onset of depression, being a woman)^9–14^. Some of the few available studies about genetic risk factors of TESI and TWOSI reported associations with genes involved in the neurotrophic and synaptic plasticity systems (*BDNF*, *NTRK2*, and *CREB1*)^15–17^, noradrenergic system (*ADRA2A*)^16^, glutamatergic system (*GRIA3*, *GRIK2*, and *GDA*)^18–20^, stress and inflammatory responses (*FKBP5* and *IL28RA*)^21,22^, opioid system (*OPRM1*)^23^, and glycoprotein synthesis (*PAPLN*)^22^.

Moreover, it is acknowledged that elements of the hypothalamic-pituitary-adrenal (HPA) axis are implicated in SB^24,25^. For instance, dexamethasone resistance predicts the risk of future suicide in patients with mood disorders^26^. Other studies showed that SB is associated with HPA axis overactivity and with excessive cortisol response to stress^27^. One proposed explanation is that the glucocorticoid receptor (GR) feedback inhibition is impaired in these patients^28^, possibly due to expression deregulation or dysfunction of HPA axis genes due to single nucleotide polymorphisms (SNPs) or epigenetic variations. Some HPA axis genes were previously described and studied in MDD and also in SB, particularly Nuclear Receptor subfamily 3, group C, member 1 (*NR3C1*) that encodes GR^29^, *FKBP5* that encodes the 51 kDa FK506 binding protein 5 (a GR-related chaperone protein)^30^, *AVPR1B* that encodes arginine vasopressin receptor 1B located in the anterior pituitary and responsible of adreno-cortico-trophic hormone (ACTH) release^31^, *CRHR1* that encodes corticotropin-releasing hormone receptor 1 which binds to neuropeptides of the corticotropin-releasing hormone family^32^, and *SKA2* that encodes spindle and kinetochore associated complex subunit 2 involved in GR transport into the nucleus and essential for chromosome segregation during mitosis^33^. A recent study demonstrated that the *FKBP5* and *NR3C1* gene promoters are significantly hypermethylated in patients with MDD compared with healthy controls, and that this leads to a significant downregulation of these genes^34^. Moreover, *FKBP5* haplotype has been associated with the risk of SA, and *NR3C1* gene expression in the prefrontal cortex with suicide^27^. A recent study found an interaction between epigenetic changes in *CRHR1* and SA in adults and general psychiatric risk scores in adolescents^35^. *AVPR1B* also has been associated with mood disorders and SA. Ben-Efraim et al.^36^ found that *AVPR1B* genetic variation may have a role in the etiology of SA characterized by severe depression symptoms. Finally, some recent studies found a link between *SKA2* expression and post-traumatic stress disorder^37^, prefrontal cortex thickness^38^, and SB^39^. The SNP rs7208505, located in the 3′UTR of *SKA2*, has two possible alleles: A (dominant) and G (recessive). Studies found that the G allele is associated with *SKA2* hypermethylation and with decreased expression in the prefrontal cortex of suicide victims^39^. Moreover, *SKA2* genetic and epigenetic variations have been associated with SA and SB^40^.

Finally, the type of antidepressant treatment also can influence TESI/TWOSI appearance. For example, in a previous study in a different cohort of outpatients with depression, we found that the risk of developing TWOSI was lower in patients receiving tianeptine than other antidepressant drugs (e.g., selective serotonin reuptake inhibitor)^41^. This could be linked to the drug mechanism of action. Indeed, tianeptine is a mu-opioid receptor (MOR) agonist^42^), and growing evidences suggest that the opioid system is deregulated in patients with SB^43^. Moreover, the opioid system modulates the HPA axis activity^44,45^ by increasing (MOR antagonist)^46^ and decreasing its activation (MOR agonist)^47^. Thus, it would be important to assess the association between TESI/TWOSI and SNPs in genes of the HPA axis in patients taking tianeptine because of the interactions between HPA axis and the opioid system. In this study, we determined whether SNPs in five HPA axis genes (*n* = 7 SNPs for *NR3C1*, *n* = 5 for *FKBP5*, *n* = 1 for *AVPR1B*, *n* = 1 for *CRHR1*, and *n* = 1 for *SKA2*) were associated with TESI and TWOSI in a large population of outpatients with MDD and treated with tianeptine.

## Materials and methods

### Participants and clinical assessment

GENESE is a large, prospective, naturalistic cohort of 3771 French outpatients with a diagnosis of a major depressive episode (MDE) and treated with tianeptine. Only 3566 patients were included in the present analyses: 120 did not meet all inclusions criteria and 85 had missing data for primary variables. The tianeptine dosage was chosen by their general practitioner (GP) or psychiatrist, and ranged between 12.5 and 37.5 mg/d, according to the prescription recommendations. Tianeptine was prescribed as first antidepressant medication or as a change of antidepressant molecule. Other concomitant treatments for current somatic problems or depression-associated symptoms (e.g., sleep and agitation) were permitted, based on clinical judgment. The same physician followed patients for at least 6 weeks between the first and second visit. At the first visit, physicians validated the MDE diagnosis according to the DSM-IV criteria and recorded sociodemographic and clinical data (e.g., lifetime SA). Concerning the patients’ history of SB, only history of lifetime SA was assessed (no information on lifetime SI, for example). Exclusion criteria were: patient younger than 18 years, non-Caucasian ethnicity, alcohol and substance dependence, and psychiatric pathology from axis I other than current MDE.

The study was performed according to the French regulatory guidelines and the current Good Clinical Practice codes. Each patient was informed about the study aims and procedures and provided a written, signed consent. The study protocol was submitted to and approved by local independent ethic committees (Comité de Protection des Personnes CPP Ile de France XI-CPPIDF11, Centre Hospitalier Intercommunal CHI Poissy Saint-Germain, Saint Germain en Laye, reference no. 08042).

Depression severity was assessed with the French version of the Hospital Anxiety and Depression Scale (HADS) completed by the physician at baseline and week 6, and by the patient at baseline, week 2, 4, and 6. Most factor analyses found a two-factor solution in accordance with the Anxiety (HADS-A) and Depression (HADS-D) subscales^48^. This scale was chosen for its simplicity of use and for its good psychometric properties^49^. SI was assessed with item 10 of the Montgomery-Åsberg Depression Rating Scale (MADRS-SI) completed by the patient at baseline, week 1, 2 and 6, and by the physician at baseline and at week 6. The rating ranges from 0 to 6: 0 to (1) enjoys life or takes it as it comes; (2–3) weary of life, only fleeting suicidal thoughts; (4–5) probably better off dead, suicidal thoughts are common, and suicide is considered as a possible solution, but without specific plans or intention; and (6) explicit plans for suicide when there is an opportunity, active preparations for suicide. A single suicide item from a depression rating scale, either clinician-rated or self-reported, is a valid approach to assess SI compared with the Beck Scale for Suicidal Ideation^50^. This method was previously used in large clinical studies, such as the STAR*D^14^, and also in more recent studies^10,41,51^.

Depression severity and SI were assessed by the physicians at baseline and at the follow-up end and by the patients at baseline, and then week 2, 4, and 6. Our priority was to collect self-report data because patients are more likely to disclose SI in self-reported measures than to a clinician, and self-report seems to be a good predictor of future SA^52,53^. Moreover, longitudinal depression and SI monitoring were needed for our study.

### SNP selection

SNPs were selected according to four criteria: (1) SNPs in genes of the HPA axis that were previously reported to be associated with suicide or depression, (2) minor allele frequencies >5%, (3) distance between SNPs >10 000 bp to limit linkage disequilibrium, and (4) total SNP number <20 because of the moderate sample size. Finally 16 SNPs were included: rs878886 in *CRHR1*; rs7208505 in *SKA2*; rs3800373, rs7757037, rs737054, rs1360780, rs9470080, rs6902321 in *FKBP5*; rs28632197 in *AVPR1B*; rs33388, rs4912905, rs2963155, rs41423247, rs6189, rs4607376 and rs12656106 in *NR3C1*. The Hardy-Weinberg equilibrium was respected for all SNPs.

### Genotyping

DNA was extracted from buccal swab samples collected at baseline. Genotyping was performed using the 5′ exonuclease assay (TaqMan, Life Technologies) and the Applied Biosystem 7900HT Fast Real-Time PCR System (Life Technologies).

The success rate of SNP genotyping in our population was 95%. In each 96-well plate, four DNA samples were assessed in replicate (duplicate) to measure the reproducibility rate. When a discrepancy in one of these replicates was observed, then the plate was genotyped again. Overall, the replication rate was 99.9% for all genotyping runs.

### Definition of TESI and TWOSI

TESI is the appearance of SI in patients without SI at baseline (i.e., at treatment initiation with a new antidepressant drug), as defined in the previous studies^15^. Therefore, the risk of TESI was evaluated only in the subsample of patients with baseline MADRS-SI score ≤1. TESI was defined as having a MADRS-SI score of 0 or 1 at baseline, followed by a score >1 at least once during the follow-up (week 2, 4, and 6) (*N* = 112 patients). Patients without TESI had a MADRS-SI score ≤1 throughout the study (baseline, week 2, 4, and 6) (*N* = 384).

TWOSI is the worsening of pre-existing (i.e., baseline) SI when starting a new antidepressant drug, as defined in the previous studies^14^. Consequently, the risk of TWOSI was evaluated only in the subsample of patients with baseline MADRS-SI score >1. TWOSI was defined by an increase of at least one point in the MADRS-SI score during the follow-up compared with baseline (*N* = 319). The group without TWOSI included all patients whose MADRS-SI score did not increase during the follow-up (*N* = 2209).

**Fig. 1:**
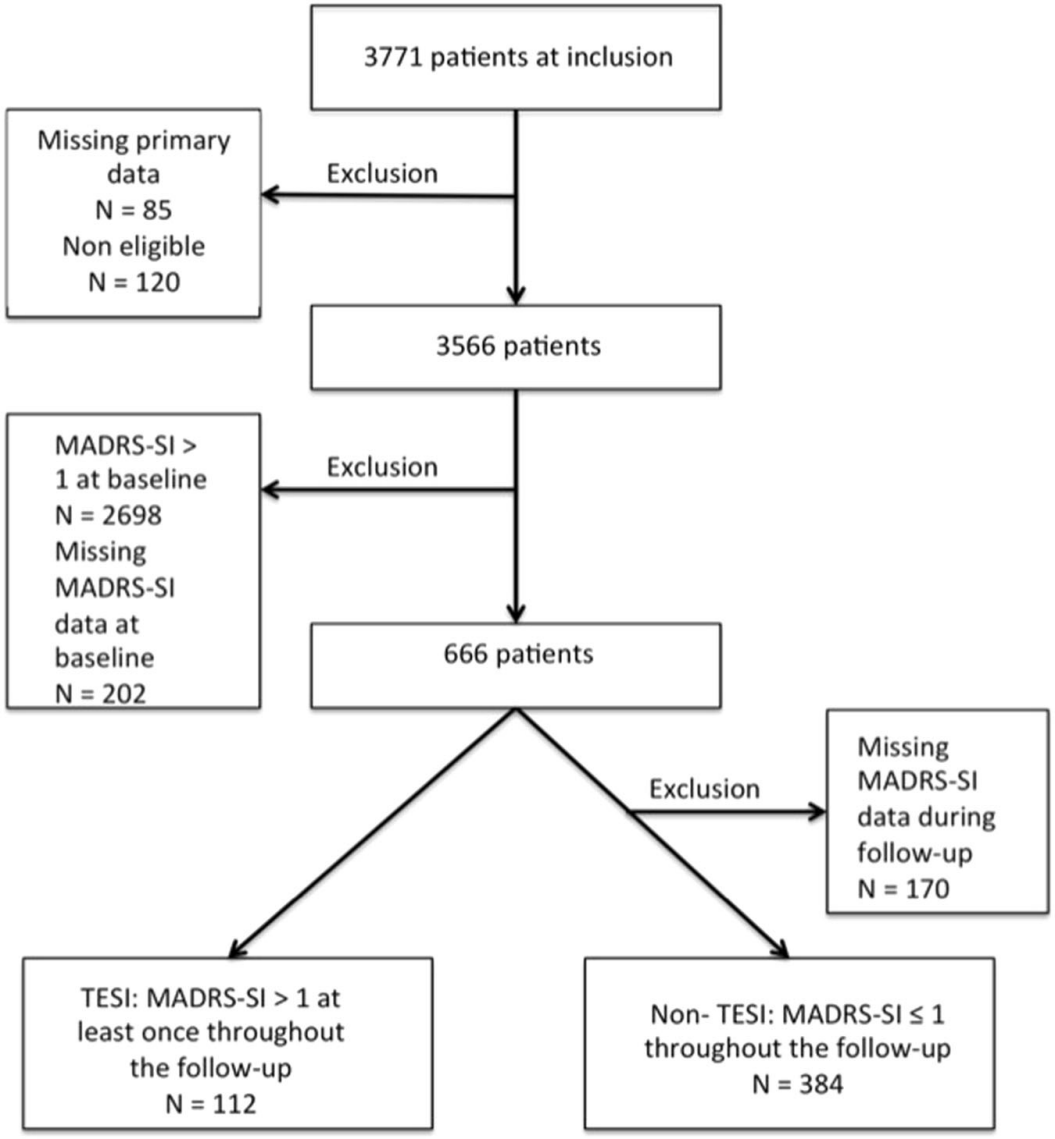
Selection of patients for TESI. This is the flowchart describing the selection of patients for treatment-emergent suicidal ideation (TESI) analysis. TESI was defined as having a MADRS-SI (item suicidal ideation of the MADRS) score of 0 or 1 at baseline, followed by a score > 1 at least once during the follow-up (week 2, 4, and 6) (*N* = 112 patients). Patients without TESI had a MADRS-SI score ≤ 1 throughout the study (baseline, week 2, 4 and 6) (*N* = 384).

**Fig. 2:**
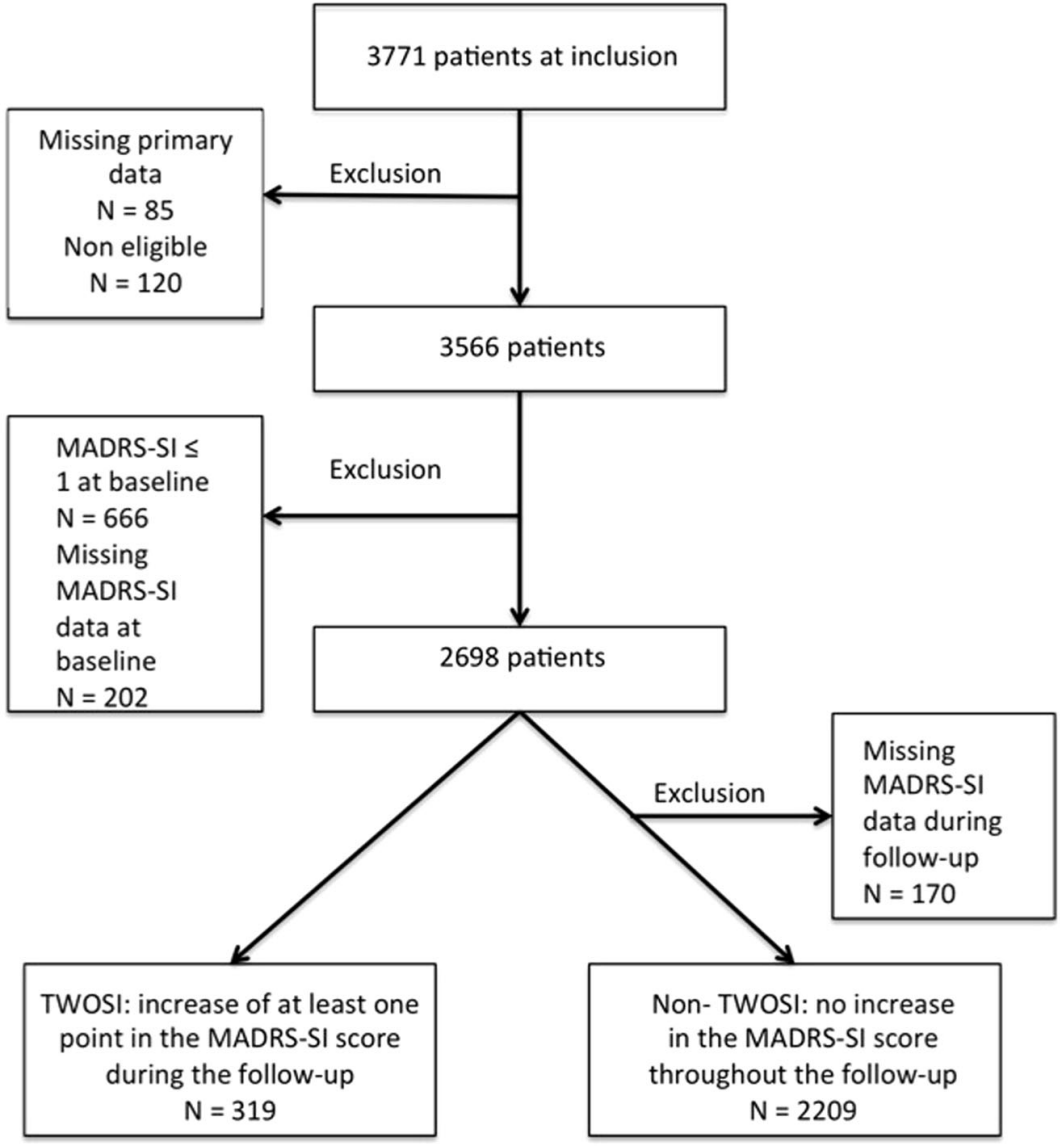
Selection of patients for TWOSI. This is the flowchart describing the selection of patients for treatment worsening suicidal ideation (TWOSI) analysis. TWOSI was defined by an increase of at least one point in the MADRS-SI (item suicidal ideation of the MADRS) score during the follow-up compared with baseline (*N* = 319). The group without TWOSI included all patients whose MADRS-SI score did not increase during the follow-up (*N* = 2209).

For the non-TESI/TWOSI groups, patients with one or more missing values during the follow-up were excluded from the analysis to include only real non-TESI/TWOSI patients. The patients’ selection flowcharts are presented in Fig. 1 (TESI) and Fig. 2 (TWOSI).

### Statistical analyses

Categorical variables were presented as percentages, and quantitative variables as means with standard deviation (SD). Demographic and clinical characteristics between patients’ groups were analyzed using a univariate logistic regression model. Baseline sociodemographic and clinical variables associated with the outcome (*p* < 0.10) were included in the first multivariate logistic regression model to estimate the adjusted odds ratios (OR) and 95% confidence intervals (CI). Furthermore, the depression severity change (HADS final total score – HADS baseline total score) was calculated and incorporated in the second multivariate logistic regression model to evaluate associations independently of depression course. The global *p*-value was computed using the Wald test obtained for the logistic regression analysis results. The sample size of GENESE of 3566 adult outpatients was sufficient to detect a difference of 5% between two groups. The significance level was set at *P* < 0.05. Analyses were performed using the SPSS statistical software (version 23.0.0.2; IBM SPSS Statistics for Windows. Armonk, NY: IBM Corp). The Bonferroni correction for multiple comparisons was implemented in all three analyses, but not for the five SNPs because they were considered independent analyses, and consequently significant with *p* < 0.05/3 = <0.016. Nevertheless, a restrictive correction of the *p*-value for all three analyses and five SNPs indicated that a corrected *p* < 0.0033 should be considered significant.

## Results

### Sociodemographic and baseline clinical characteristics associated with TESI

At baseline, 496 patients had a MADRS-SI score of 0–1. Their mean age was 48.05 years (SD = 14.75) and 38.7% were men. Among these patients, 112 had a MADRS-SI score ≥2 at least once during the follow-up (TESI group) and 384 had a MADRS-SI score of 0–1 throughout the study (non-TESI group). Compared with the non-TESI group, patients in the TESI group were more frequently men (*p* = 0.01), with benzodiazepine co-prescription (*p* = 0.01), and alcohol abuse during follow-up (*p* = 0.02). They also tended to have lifetime SA (*p* = 0.06), and to have had previous pharmacological treatments for depression (*p* = 0.05) (Table 1). Therefore, the first multivariate logistic regression models were adjusted for these factors: sex, lifetime SA, treatment instauration, alcohol abuse, and benzodiazepine intake. In the second multivariate logistic regression models, change in depression scores was added to these adjustment variables.

**Table 1:** Association between sociodemographic and clinical characteristics and treatment-emergent suicidal ideation (TESI).

	TESI				
	No		Yes		
	*N* = 384		*N* = 112		
Variables	*N*	%	*N*	%	*P-*value
Sex					**0.01**
Men	137	35.7	55	49.1	
Women	247	64.3	57	50.9	
Age (years)^a^	47.84 (14.85)	48.76 (14.47)			0.56
Marital status					0.95
Single	75	19.6	20	17.9	
Married	232	60.7	68	60.7	
Divorced	54	14.1	18	16.1	
Widower	21	5.5	6	5.4	
Study level					0.22
Below secondary school diploma	144	38	52	47.3	
Secondary school diploma	104	27.4	26	23.6	
University	131	34.6	32	29.1	
Professional activity					0.46
Working	232	61.4	64	57.7	
Unemployment	21	5.6	10	9	
Retired	71	18.8	18	16.2	
Other	54	14.3	19	17.1	
MDE duration					
<2 months	144	37.5	41	36.6	0.12
[2; 6] months	153	39.8	36	32.1	
>6 months	83	21.6	34	30.4	
First MDE					0.12
No	143	37.3	51	45.5	
Yes	240	62.7	61	54.5	
Number of MDE^a^	2.46 (1.84)		2.21 (0.98)		0.91
Age at first MDE (years)^a^	36.38 (15.33)		36.33 (14.18)		0.98
Baseline HADS-D^a^	12.24 (3.88)		12.08 (3.92)		0.69
Baseline HADS-A^a^	12.89 (3.37)		12.61 (3.52)		0.45
Baseline HADS total score^a^	25.13 (6.07)		24.69 (6.08)		0.98
Lifetime suicide attempts					**0.06**
No	362	96.5	99	91.7	
Yes	13	3.5	9	8.3	
Benzodiazepine					**0.01**
No	217	56.7	47	43.1	
Yes	166	43.3	62	56.9	
Alcohol abuse					**0.02**
No	372	98.2	99	93.4	
Yes	7	1.8	7	6.6	
Treatment instauration					**0.05**
No	53	13.8	24	21.6	
Yes	330	86.2	87	78.4	

*MDE* Major Depressive Episode, *HADS* Hospital Anxiety and Depression Scale.

^a^Continuous variables were expressed as mean (standard deviation).

Bold values indicates significant *p*-values (*p*-value < 0.05).

### SNPs association with TESI

Four SNPs were significantly associated with TESI, one in *NR3C1*, two in *FKBP5*, and one in *SKA2*. No SNP in *CRHR1* and *AVPR1B* was associated with TESI. The AG phenotype of the SNP rs2963155 in *NR3C1* was associated with TESI in the crude analysis (OR = 1.87, 95% CI = [1.16; 3.01]; *p*-value = 0.03; model 0, Table 2). However, when potential confounders were entered in the model, the OR decreased (OR = 1.76, 95% CI = [1.05; 2.94] for the AG genotype; *p*-value = 0.05; model 1, Table 2) and the association was not significant after adjustment also for depression severity changes (*p*-value=0.12; model 2, Table 2). The TT genotype of the SNP rs737054 in *FKBP5* was significantly associated with TESI in the crude analysis and also after adjustment (OR = 2.36, 95% CI = [1.04; 5.32]; *p*-value = 0.04; model 1, Table 2), except when adjusted for depression severity changes (*p*-value = 0.06; model 2, Table 2). The TT genotype of the SNP rs6902321 in *FKBP5* tended to be associated with TESI (*p*-value = 0.09), and this trend became significant after adjusting for potential cofounders (OR = 1.66, 95% CI = [1.02; 2.69]; *p*-value = 0.04; model 1, Table 2). This association was stronger after adjustment for depression severity changes (OR = 1.76, 95% CI = [1.07; 2.90]; *p*-value = 0.03; model 2, Table 2). Finally, the trend for an association between the GG/AG genotype of rs7208505 in *SKA2* and TESI in the unadjusted analysis (OR = 1.65, 95% CI = [0.97; 2.80]; *p*-value = 0.07; model 0, Table 2) became significant after adjustment for potential cofounders (OR = 1.81, 95% CI = [1.02; 3.19]; *p*-value = 0.04; model 1, Table 2) and also for depression severity changes (OR = 1.85, 95% CI = [1.03; 3.33]; *p*-value = 0.04; model 2, Table 2). None of these associations remained significant after the Bonferroni correction for multiple tests.

**Table 2:** Associations between SNPs of genes implicated in the HPA axis and treatment-emergent suicidal ideation (TESI).

	TESI									
	No (*n* = 384)		Yes (*n* = 112)		Model 0		Model 1		Model 2	
	*n*	%	*n*	%	OR [95% CI]	*P*-value	OR [95% CI]	*P*-value	OR [95% CI]	*P-*value
***NR3C1*** **rs2963155**										
** AA/AG**	306	92.4	91	94.8	1	0.43	1	0.26	1	0.38
** GG**	25	7.6	5	5.2	0.67 [0.25; 1.81]		0.53 [0.17; 1.63]		0.60 [0.19; 1.86]	
** AA**	**207**	**62.5**	**48**	**50**	**1**	0.03^*^	1	0.09	1	0.14
** GG/AG**	**124**	**37.5**	**48**	**50**	**1.67 [1.06; 2.64]**		0.66 [0.4; 1.08]		0.68 [0.41; 1.14]	
rs33388										
AA/AT	254	78.2	70	73.3	1	0.36	1	0.21	1	0.24
TT	71	21.8	25	26.3	1.28 [0.75; 2.16]		1.42 [0.82; 2.48]		1.41 [0.79; 2.49]	
TT/AT	233	71.7	65	68.4	1	0.54	1	0.72	1	0.91
AA	92	28.3	30	31.6	1.17 [0.71; 1.92]		1.10 [0.65; 1.88]		1.03 [0.60; 1.78]	
rs4912905										
GG/CG	315	96.3	90	94.7						
CC	12	3.7	5	5.3						
CC/CG	123	37.6	37	38.9	1	0.81	1	0.40	1	0.43
GG	204	62.4	58	61.1	0.95 [0.59; 1.51]		0.80 [0.49; 1.33]		0.81 [0.48; 1.37]	
rs41423247										
GG/GC	283	88.4	77	81.1	1	0.07	1	0.19	1	0.12
CC	37	11.6	18	18.9	1.78 [0.96; 3.31]		1.55 [0.80; 3.01]		1.70 [0.87; 3.34]	
CC/CG	192	60	59	62.1	1	0.71	1	0.66	1	0.60
GG	128	40	36	37.9	0.92 [0.57; 1.47]		0.89 [0.54; 1.48]		0.87 [0.52; 1.46]	
rs6189										
GG/GA	330	99.7	99	100						
AA	1	0.3	0	0						
AA/AG	13	3.9	4	4						
GG	318	96.1	95	96						
rs4607376										
GG/GA	265	80.3	76	78.4	1	0.67	1	0.76	1	0.37
AA	65	19.7	21	21.6	1.13 [0.65; 1.96]		0.91 [0.49; 1.68]		0.74 [0.38; 1.43]	
AA/AG	231	70	71	73.2	1		1	0.76	1	0.78
GG	99	30	26	26.8	0.85 [0.52; 1.42]		1.09 [0.63; 1.87]		1.08 [0.62; 1.88]	
rs12656106										
GG/GC	266	81.8	74	77.9	1	0.39	1	0.46	1	0.90
CC	59	18.2	21	22.1	1.28 [0.73; 2.24]		1.25 [0.69; 2.27]		1.04 [0.56; 1.95]	
CC/GC	198	60.9	61	64.2	1		1	0.93	1	0.99
GG	127	39.1	34	35.8	0.87 [0.54; 1.39]		0.98 [0.59; 1.63]		1.00 [0.59; 1.68]	
***FKBP5*** **rs737054**										
**CC/CT**	**308**	**93.6**	**85**	**86.7**	**1**	0.03^*^	**1**	0.04^*^	**1**	0.06^*^
**TT**	**21**	**6.4**	**13**	**13.3**	**2.24 [1.08; 4.67]**		**2.36 [1.04; 5.32]**		**2.24 [0.97; 5.15]**	
**TT/CT**	150	45.6	47	48	1	0.68	1	0.65	1	0.55
**CC**	179	54.4	51	52	0.91 [0.58; 1.43]		0.89 [0.55; 1.46]		0.86 [0.52; 1.42]	
**rs6902321**										
**TT/CT**	305	89.4	91	91	1	0.65	1	0.55	1	0.44
**CC**	36	10.6	9	9	0.84 [0.39; 1.80]		0.77 [0.33; 1.83]		0.70 [0.29; 1.71]	
**CC/CT**	**186**	**54.8**	**45**	**45**	**1**	0.09^*^	**1**	0.04^*^	**1**	0.03^*^
**TT**	**154**	**45.2**	**55**	**55**	**1.48 [0.95; 2.32]**		**1.66 [1.02; 2.69]**		**1.76 [1.07; 2.90]**	
rs3800373										
TT/TG	282	90.4	85	91.4	1	0.77	1	0.53	1	0.47
GG	30	9.6	8	8.6	0.88 [0.39; 2.00]		0.75 [0.29; 1.89]		0.70 [0.27; 1.81]	
GG/TG	159	51	42	45.2	1	0.33	1	0.36	1	0.32
TT	153	49	51	54.8	1.26 [0.79; 2.01]		1.26 [0.77; 2.08]		1.30 [0.78; 2.17]	
rs7757037										
GG/AG	261	79.6	72	74.2	1	0.26	1	0.38	1	0.46
AA	67	20.4	25	25.8	1.35 [0.80; 2.29]		1.30 [0.72; 2.36]		1.26 [0.68; 2.32]	
AA/AG	229	69.8	68	70.1	1	0.96	1	0.99	1	0.82
GG	99	30.2	29	29.9	0.99 [0.60; 1.62]		1.00 [0.59; 1.71]		0.94 [0.54; 1.63]	
rs1360780										
CC/CT	292	89.6	90	91.8	1	0.51	1	0.34	1	0.34
TT	34	10.4	8	8.2	0.76 [0.34; 1.71]		0.64 [0.25; 1.60]		0.63 [0.25; 1.61]	
TT/CT	175	53.7	46	46.9	1	0.24	1	0.13	1	0.09
CC	151	46.3	52	53.1	1.31 [0.83; 2.06]		1.46 [0.89; 2.40]		1.54 [0.93; 2.57]	
rs9470080										
CC/CT	286	87.7	87	90.6	1	0.44	1	0.36	1	0.29
TT	40	12.3	9	9.4	0.74 [0.35; 1.59]		0.67 [0.29; 1.58]		0.63 [0.26; 1.49]	
TT/CT	184	56.4	48	50	1	0.27	1	0.21	1	0.15
CC	142	43.6	48	50	1.30 [0.82; 2.05]		1.36 [0.84; 2.23]		1.45 [0.88; 2.40]	
***SKA2*** **rs7208505**										
AA/AG	217	86.5	65	81.3	1	0.25	1	0.26	1	0.39
GG	34	13.5	15	18.8	1.47 [0.76; 2.87]		1.52 [0.74; 3.16]		1.40 [0.65; 3.01]	
** AA**	**111**	**44.2**	**26**	**32.5**	**1**	0.07^*^	**1**	0.04^*^	**1**	0.04^*^
**GG/AG**	**140**	**55.8**	**54**	**67.5**	**1.65 [0.97; 2.80]**		**1.81 [1.02; 3.19]**		**1.85 [1.03; 3.33]**	
***CRHR1*** rs878886										
CC/CG	312	95.7	92	94.8						
GG	14	4.3	5	5.2						
GG/CG	139	42.6	48	49.5	1	0.23	1	0.22	1	0.22
CC	187	57.4	49	50.5	0.76 [0.48; 1.19]		0.73 [0.45; 1.20]		0.73 [0.44; 1.21]	
***AVPR1B*** rs28632197										
GG/AG	319	99.1	93	100						
AA	3	0.9	0	0						
AA/AG	64	19.9	14	15.1	1	0.30	1	0.24	1	0.16
GG	258	80.1	79	84.9	1.40 [0.75; 2.63]		1.50 [0.76; 2.95]		1.66 [0.82; 3.36]	

Model 0: Crude association.

Model 1: Adjusted for sex, lifetime suicide attempt, treatment instauration, alcohol abuse, and benzodiazepine intake.

Model 2: Adjusted for sex, lifetime suicide attempt, treatment instauration, alcohol abuse, benzodiazepine intake, and depression severity change.

Bold values indicates significant *p-values* (*p*-value  <  0.05).

*Bonferroni correction applied (a corrected *p* < 0.016 was considered as significant).

### SNP association with TWOSI

At baseline, 2528 patients had a MADRS-SI ≥ 2. They mean age was 49 years (SD = 14.65), and 38.9% were men. Among them, 319 (12.6%) met the criteria for TWOSI according to our definition. None of the tested SNPs was associated with TWOSI (data not shown).

## Discussion

To our knowledge, this is the first study that assessed possible associations between SNPs in the *NR3C1*, *CRHR1*, *AVPR1B*, *SKA2* and *FKBP5* genes and TESI or TWOSI in a large cohort of adult outpatients with depression and treated with tianeptine (a mu opioid receptor agonist). The only exception is the TORDIA trial, but this study concerned adolescents with depression and had different outcomes (association between SNPs and symptom improvement and suicide occurrence)^21^.

We found that there was a trend for an association between TESI and the SNP rs7208505 (AG/GG genotypes) in *SKA2* and rs6902321 (TT genotype) in *FKBP5*. These associations became significant after adjustment for potential cofounders, suggesting a strong link between these SNPs and TESI. Moreover, these associations remained significant even after adjustment for depression severity changes (and became even stronger for rs6902321), suggesting a role of these SNPs in TESI, independently of depression course. The finding for rs7208505 is in line with previous results that associated this SNP with completed suicide, SB and SA^38–40^. Indeed, the presence of the G allele of this SNP allows the creation of a CpG site, and the possible methylation of SKA2. This CpG site is associated with reduced SKA2 expression that might lead to decreased prefrontal cortex area thickness and disturbances in GR transport into the nucleus^37^. To our knowledge, there is no study on the biological consequences of the intronic SNP rs6902321 in *FKBP5*, and on its association with SB. However, other SNPs in *FKBP5* that were previously associated with suicide or SB (rs1360780 and rs3800373)^54^ were not associated with TESI in our study. This might suggest a TESI-specific physiopathology.

The association between TESI and the SNPs rs737054 (TT genotype) in *FKBP5* and rs2963155 (GG/AG genotypes) in *NR3C1* did not remain significant after adjustment for depression severity changes. This suggests that there is an interaction between these SNPs, TESI, and depression changes. Concerning the intronic SNP rs2963155 in *NR3C1*, the presence of the G allele might affect alternative splicing and consequently it might have an impact on GR function and/or expression. Additional studies are needed to evaluate its effect on GR function^55,56^. Finally, although the functional consequences of the intronic SNP rs737054 are unknown, it has been associated with other psychiatric disorders, such as borderline personality disorders^57^.

Unfortunately, when the Bonferroni correction was applied, no association remained significant. However, it is acknowledged that the Bonferroni correction is conservative, particularly when the sample size is small (like in our study) and multiple tests are performed, and might lead to false-negative results^58^. Moreover, our TESI phenotype was precisely and strictly defined, all selected genes were previously associated with SB, and our hypothesis was well established, thus strengthening our findings. In conclusion, our results suggest that the *NR3C1*, *FKBP5* and *SKA2* genes of the stress pathway might be implicated in TESI. Furthermore, it is interesting to note that: (i) the SNPs associated with TESI remained significant in the first model that was adjusted for lifetime SA. Therefore, these SNPs could be involved in TESI independently of the risk of lifetime SA; (ii) the three genes with TESI-associated SNPs are all directly linked to GR function and/or transport, suggesting that impairment of GR function and/or transport could be implicated in TESI physiopathology.

These results are interesting because potential news drugs are emerging for treatment-resistant depression and SB. For instance, ketamine (an NMDA antagonist) seems to be a promising molecule for the management of “suicidal crisis” (i.e. depression with severe SI)^59^, and has been recently authorized by the US FDA for treatment-resistant depression^60^. Its efficacy in patients with treatment-resistant depression and SI could be explained by its action on the glutamatergic system, known to interact with the HPA axis^61,62^. It could be interesting to study the interaction between ketamine, HPA axis genes and TESI. Finally, it is important to note that all patients in our cohort were taking tianeptine, a MOR agonist^42^. As the opioid system might interact with the HPA axis and the response to stress^63,64^, results could have been different in patients taking other types of antidepressant drugs.

This study has some limitations. First, the size of the TESI group was relatively small, despite the very large cohort sample, due to the rarity of this phenomenon. Moreover, there is no consensus on the definition of TESI. In this study, we chose to favor specificity by including in the non-TESI group all patients who switched between the MADRS-SI scores of 0 and 1. Second, due to the study design only outpatients with MDE were included, and therefore patients with the most severe disease, perhaps more inclined to develop TESI, might have been excluded. Finally, the small number of patients with the AA genotype of rs28632197 in *AVPR1B* and with the AA/AG genotype of rs6189 in *NR3C1* led sometimes to inconclusive results.

In conclusion, our results contribute to our knowledge on the HPA axis implication in SB, specifically in TESI. More studies are needed to better understand the mechanisms of HPA axis implication in SB and TESI in order to find new therapeutic targets and prevent SB.
